# Comparing Kangaroo Mother Care and Expressed Breast Milk as Non-pharmacological Strategies for Reducing Procedural Pain in Neonates: A Randomized Controlled Trial

**DOI:** 10.7759/cureus.86423

**Published:** 2025-06-20

**Authors:** Santosh K Lodhi, Suman Rana, Surendra Kumar, Preeti Kumari, Narasimha Rao, Kasi Raghava, Brajendra Singh, Karnika Agrawal, Rakesh Kumar

**Affiliations:** 1 Pediatrics, Maharaja Agrasen Medical College, Agroha, Hisar, IND; 2 Obstetrics and Gynaecology, Maharaja Agrasen Medical College, Agroha, Hisar, IND

**Keywords:** expressed breast milk, kangaroo mother care, neonatal intensive care unit, neonatal pain, neonates, non-pharmacological pain management, premature infant pain profile

## Abstract

Background: Neonates in neonatal intensive care units frequently endure painful procedures, such as adhesive tape removal, often without adequate pain management. Non-pharmacological interventions like kangaroo mother care (KMC) and expressed breast milk (EBM) administration have shown promise in alleviating neonatal pain. However, comparative data on their efficacy for specific procedures are limited.

Objective: This study compares the effectiveness of KMC and EBM in reducing procedural pain during adhesive tape removal in low-birth-weight neonates.

Methods: In this randomized controlled trial conducted at a tertiary care neonatal intensive care unit, 100 low-birth-weight neonates were randomized into KMC and EBM groups. Procedural pain during adhesive tape removal was assessed using the Premature Infant Pain Profile (PIPP) score, incorporating heart rate (HR), oxygen saturation (SpO₂), and facial expressions pre- and post procedure. Video recordings were analyzed by a single trained assessor to ensure reliability.

Results: The baseline characteristics of both groups were comparable. Post-procedure PIPP scores were slightly lower in the KMC group (11.02 ± 2.39) than the EBM group (11.19 ± 2.22), but the difference was not statistically significant (p = 0.704). HR, SpO₂, and facial expression parameters were also similar between groups. Difference in pre- and post-procedure PIPP scores in KMC (4.41 ± 1.72) and the EBM group (4.04 ± 1.50) was also comparable (p = 0.275).

Conclusion: KMC and EBM are equally effective, low-cost, and accessible strategies for procedural pain management in neonates. This study highlights the importance of integrating non-pharmacological pain relief methods into routine neonatal care.

## Introduction

Neonates admitted to neonatal intensive care units (NICUs) frequently undergo invasive diagnostic and therapeutic procedures that are often associated with significant pain, such as cannulation, endotracheal intubation, heel pricks, and adhesive tape removal. Pain management for neonates remains inconsistent globally, with studies indicating that up to 80% of neonates in NICUs receive inadequate pain relief during routine procedures [1,2]. This highlights the need for effective pain management strategies in this vulnerable population.

The perception of pain in neonates is well-established. By 20 weeks of gestation, peripheral nerve fibers are fully developed, and by 28-30 weeks, nociceptive nerve endings achieve adult-like densities [3]. Neonatal pain triggers a cascade of physiological responses, including increased oxygen demand, alterations in cerebral blood flow, and stress hormone release. Long-term effects include heightened pain sensitivity, behavioral issues, and emotional dysregulation in later life [4,5]. Addressing neonatal pain is both an ethical imperative and a human right, emphasizing the necessity for effective interventions [6,7].

Non-pharmacological strategies have emerged as viable solutions to minimize pain without the adverse effects of medications. Kangaroo mother care (KMC) involves direct skin-to-skin contact, which promotes the release of endorphins and reduces cortisol levels, enhancing thermal regulation, breastfeeding success, and mother-infant bonding [8,9]. Expressed breast milk (EBM) exerts analgesic effects due to its sweetness (presence of lactose and other components) and the presence of bioactive components such as tryptophan, a precursor of melatonin, which enhances beta-endorphin release [10-13]. Both are natural, readily available, and potentially risk-free interventions for neonatal pain relief.

Adhesive tape removal was selected as the procedure for this study due to its routine nature and its potential to cause considerable discomfort in neonates with fragile skin, often leading to microtears and heightened nociceptive responses. Despite the availability of both KMC and EBM, comparative data on their efficacy in procedural pain management during tape removal are sparse.

This study aims to fill this gap by comparing the effectiveness of KMC and EBM in mitigating procedural pain during adhesive tape removal in low-birth-weight preterm neonates. The findings aim to inform clinical practices, promoting safe and accessible pain management strategies in NICUs worldwide.

This study aims to fill the existing gap by comparing the effectiveness of KMC and EBM in mitigating procedural pain during adhesive tape removal in low-birth-weight preterm neonates. The primary objective was to compare the post-procedural Premature Infant Pain Profile (PIPP) scores between the two groups. The secondary objective was to compare the difference between pre- and post-procedural PIPP scores within each group. The findings are intended to inform and enhance clinical practices by promoting safe, feasible, and accessible non-pharmacological pain management strategies in NICUs globally.

This article was previously presented as an abstract at the HARCON 2023 Annual Conference of the Indian Academy of Pediatrics, Haryana.

## Materials and methods

Study design

This was a double-arm, open-label, active-controlled, parallel-group randomized controlled trial.

Study setting

The study was conducted from January 2023 to June 2023 in the neonatal intensive care unit of a tertiary care hospital in Haryana, India. Ethical clearance was obtained from the Institutional Ethical Committee (Reference number: MAMC/IEC/2022/28). The trial was registered in the Clinical Trials Registry-India (CTRI) with registration number CTRI/2023/06/053402.

Sample size

The sample size was calculated for this non-inferiority trial comparing PIPP scores between neonates receiving KMC and those given EBM during adhesive tape removal using OpenEpi, an open-source, web-based software developed by the Centers for Disease Control and Prevention (CDC, Atlanta, GA). Considering the post-procedure mean PIPP score of 12.13 with a standard deviation of 2.59 from the study done by Nanavati et al. [14], a non-inferiority margin of 1.2, a one-sided alpha of 0.05, and power of 90%, the required sample size was 44 neonates per group. To account for possible dropouts, we inflated the sample size by 10%.

Study population

Sequentially born, low-birth-weight preterm neonates admitted to the NICU regardless of their initial diagnosis but who were clinically improving and hemodynamically stable, were recruited for the study. The inclusion criteria comprised preterm neonates of both genders with a gestational age of less than 37 weeks and a birth weight between 1500 grams and 2500 grams who were hemodynamically stable. Neonates were excluded if they had congenital malformations, neurological abnormalities, or were under sedation, receiving analgesics, or on anti-epileptic medications, as these conditions can impair behavioral and physiological responses to pain and affect the reliability of pain assessment.

Method in brief

Parental informed consent was obtained for all participants. Randomization was performed using a computer-generated random number table prepared by an independent statistician, with a 1:1 allocation ratio to assign neonates to either the KMC group or the EBM group. To ensure allocation concealment, sequentially numbered opaque sealed envelopes (SNOSE) were prepared in advance by the NICU nursing in-charge, with each envelope containing the assigned intervention as per the randomization sequence. These envelopes were securely stored and opened sequentially by the principal investigator at the time of participant enrollment to determine group allocation. This was an open-label trial, and no blinding was performed due to the nature of the interventions.

Baseline characteristics, including gestational age, postnatal age, baseline oxygen saturation (SpO₂), heart rate (HR), and weight, were recorded for both groups. The NICU environment was maintained at a temperature of 25-28°C with a noise level below 35 to 40 decibels.

The PIPP score was used to evaluate pain both pre- and post procedure. It is a pain assessment tool comprising seven indicators, including three behavioral responses (brow bulge, eye squeeze, and nasolabial furrow), two physiological parameters (HR and SpO₂), and two contextual factors (gestational age and behavioral state). Each indicator is rated on a four-point scale (0 to 3), resulting in a maximum possible score of 21. The total PIPP score varies according to gestational age. For all gestational age groups, a PIPP score of ≤6 indicated minimal/no pain, while a score ≥12 indicated moderate to severe pain.

In both groups, behavioral state was assessed by observing the neonate for 15 seconds before the intervention (adhesive tape removal). The assessment parameters included infant activity, eye status (open or closed), and facial expressions. The baseline HR, SpO₂, and the three behavioral responses were also recorded. Adhesive tape was removed by the principal investigator in all participants. The tape used was hypoallergenic, latex-free, water-resistant, and made of an acrylate adhesive polymer (3M, Saint Paul, MN), measuring 2 cm in length and 1.5 cm in width, applied over the lower third of the sternum for temperature probe fixing. An independent assistant was assigned to video-record the neonate’s facial expressions during the intervention for 30 seconds for later assessment of the PIPP score. The PIPP score was later calculated by a single trained assessor by carefully observing this video, including the maximum and minimum HR and SpO₂ as per pulse oximetry reading in the recorded video.

In the KMC group, the intervention was performed after the neonate had been in skin-to-skin contact with the mother for 15 minutes and was continued during the adhesive tape removal procedure by keeping the baby in a supine position for a minute. In the EBM group, expressed breast milk was administered to the infant for two minutes prior to the tape removal procedure.

Statistical analysis

Data were collected using a structured case record form and entered into Epi Info, an open-source mobile application developed by the CDC. Categorical variables were summarized as percentages, while continuous variables were expressed as mean and standard deviation. For comparison between the two groups, the chi-square test was used for categorical variables, and the independent samples t-test was applied for continuous variables. Paired t-tests were used to analyze pre- and post-intervention data within groups. A p-value of less than 0.05 was considered statistically significant for all analyses.

## Results

**Figure 1:**
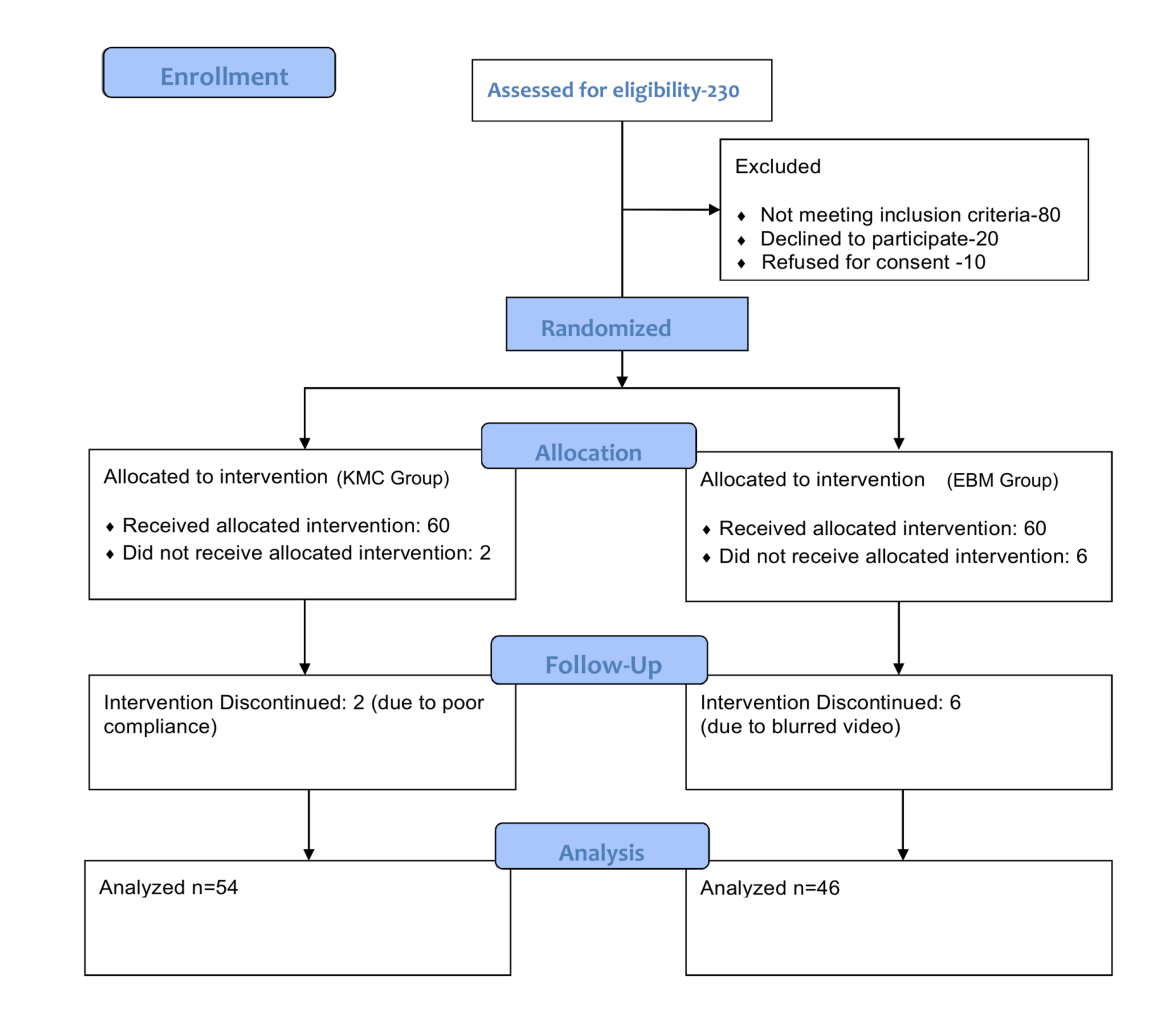
CONSORT flow diagram depicting participant enrollment, allocation, follow-up, and analysis. CONSORT: Consolidated Standards of Reporting Trials; KMC: kangaroo mother care; EBM: expressed breast milk.

Out of 230 neonates assessed for eligibility, 110 were excluded due to not meeting the inclusion criteria or declining consent. The remaining 120 neonates were randomized into two groups: 60 in the KMC group and 60 in the EBM group. After accounting for losses to follow-up, data from 54 neonates in the KMC group and 46 neonates in the EBM group were analyzed, as shown in the CONSORT flow diagram of the study (Figure 1).

The baseline characteristics of the study population, including gestational age, postnatal age, weight, gender distribution, and behavioral state score, were comparable between the KMC and EBM groups, as shown in Table 1.

**Table 1:** Comparison of baseline characteristics between study groups. KMC: kangaroo mother care; EBM: expressed breast milk; SD: standard deviation. ^# ^Independent t-test was applied; p-value considered significant if <0.05. * Chi-square test was applied; p-value considered significant if <0.05.

Parameter	KMC group (n = 54)	EBM group (n = 46)	p-value^#^
Gestational age in weeks (mean ± SD)	34.01 ± 1.18	33.71 ± 1.00	0.179
Postnatal age (days) (mean ± SD)	8.09 ± 7.46	7.63 ± 6.88	0.750
Weight (kg) (mean ± SD)	2.13 ± 0.26	2.07 ± 0.34	0.331
Female gender, n (%)	21 (38.9)	24 (52.2)	0.183^*^
Behavioral state score (mean ± SD)	0.87 ± 0.48	0.91 ± 0.28	0.620

Pre- and post-procedure parameters, including HR, SpO2, and facial expressions (brow bulge, eye squeeze, and nasolabial fold), were analyzed. Pre-procedure mean HR was marginally higher in the KMC group (134.61 bpm vs. 131.73 bpm, p = 0.080), but this was not statistically significant. Pre-procedure mean SpO2 levels were similar between the groups (p = 0.929). Post-procedure mean HR and SpO2 showed no significant differences between the groups. Facial expression indicators, including brow bulge, eye squeeze, and nasolabial fold, were comparable between groups during post-procedure assessment (p > 0.05), as presented in Table 2.

**Table 2:** Comparison of pre- and post-procedural parameters. KMC: kangaroo mother care; EBM: expressed breast milk; SD: standard deviation; HR: heart rate; SpO2: oxygen saturation. * Independent t-test was applied; p-value considered significant if <0.05.

Parameter	Phase	KMC group (mean ± SD) (n = 54)	EBM group (mean ± SD) (n = 46)	p-value^*^
HR (bpm)	Pre-procedure	134.61 ± 8.01	131.73 ± 8.20	0.080
	Post-procedure	138.07 ± 7.89	135.35 ± 9.02	0.110
SpO2 (%)	Pre-procedure	96.26 ± 1.25	96.24 ± 0.95	0.929
	Post-procedure	95.31 ± 1.02	95.13 ± 0.91	0.347
Brow bulge (score)	Pre-procedure	1.15 ± 0.63	1.26 ± 0.74	0.413
	Post-procedure	2.05 ± 0.71	2.13 ± 0.74	0.610
Eye squeeze (score)	Pre-procedure	1.15 ± 0.85	1.33 ± 0.76	0.279
	Post-procedure	2.24 ± 0.67	2.07 ± 0.74	0.217
Nasolabial fold (score)	Pre-procedure	1.38 ± 0.94	1.69 ± 0.98	0.115
	Post-procedure	2.46 ± 0.79	2.32 ± 0.70	0.367

Pain was assessed using the PIPP score. The pre-procedure mean score was comparable between the two groups (p = 0.244). The post-procedure mean PIPP score was slightly higher in the EBM group, but the difference was not statistically significant (p = 0.704). The difference between the pre- and post-procedural PIPP scores was also similar between the two groups (p = 0.275), as depicted in Table 3.

**Table 3:** Comparison of pre- and post-procedural PIPP scores in the KMC and EBM groups. KMC: kangaroo mother care; EBM: expressed breast milk; SD: standard deviation; PIPP: Premature Infant Pain Profile. * Independent t-test was applied; p-value considered significant if <0.05.

Parameter	KMC group (n = 54)	EBM group (n = 46)	p-value*
Pre-procedure PIPP score (mean ± SD)	6.57 ± 2.42	7.15 ± 2.50	0.244
Post-procedure PIPP score (mean ± SD)	11.02 ± 2.39	11.20 ± 2.23	0.704
Pre-post difference in PIPP score (mean ± SD)	4.41 ± 1.72	4.04 ± 1.50	0.275

## Discussion

Pain is a crucial physiological response in neonates, conveyed through autonomic, motor, and behavioral cues, such as changes in HR, SpO₂, and facial expressions, including brow bulge, eye squeeze, and nasolabial furrow. These indicators form the foundation of validated tools like the PIPP, which is widely employed to assess procedural pain in neonates [6,15].

Studies indicate that removing adhesive tape from newborns can be a painful procedure. The pain is often associated with the stripping of the epidermal layer when the tape is removed, potentially decreasing the barrier function of the skin. In the EPIPPAIN study, the removal of adhesives was the fourth most frequent source of presumed pain related to care in newborns hospitalized in neonatal intensive care, which represented 12.7% of the total number of painful procedures [1]. Studies have shown that pain scores increase significantly during this process [1,14]. As adhesive removal is a common and proven painful procedure in the NICU, this was chosen for our study.

Non-pharmacological interventions have emerged as effective strategies to manage minor neonatal pain without the risks associated with pharmacological treatments. Studies have demonstrated the effectiveness of KMC and EBM as non-pharmacological interventions for reducing pain in neonates. KMC, involving skin-to-skin contact, has demonstrated broad benefits, including improved thermoregulation, increased breastfeeding success, reduced mortality, and enhanced neurodevelopmental outcomes [14,16-20]. A study done by Nimbalkar et al. found PIPP scores of 5.38 ± 3.25 in the KMC group versus 10.23 ± 4.59 in the standard care (SC) group. The difference was clinically and statistically significant (p < 0.0001), which means that the perceived pain was lower in the KMC group [17]. In a clinical trial with a crossover design, KMC significantly reduced pain intensity during and after heel lancing in preterm neonates compared to prone positioning, highlighting its effectiveness as a simple, natural, and family-centered pain relief method [19]. In a Cochrane review, skin-to-skin contact (SSC) was shown to be effective, based on both composite physiological and behavioral pain indicators and, independently, using heart rate and crying time, and safe for single painful procedures [20]. However, few studies observed no significant differences in PIPP scores between KMC and standard care (SC) groups. For example, a study that assessed pain during nasal suctioning over five days in preterm infants born at 27-30 weeks gestational age revealed mild to moderate pain during suctioning, with no significant difference in pain scores between KMC and SC groups (PIPP scores of 7.64 ± 0.40 in the KMC group vs. 7.89 ± 0.21 in the SC group) [21]. These discrepancies may result from differences in study populations, procedural contexts, or intervention protocols. For example, studies employing more painful procedures, such as venipuncture or heel pricks, reported greater efficacy of KMC, while less painful procedures, like suctioning, showed diminished interventional differences.

Similarly, studies have also shown EBM to be effective for alleviating procedural pain in neonates. EBM administration serves as an accessible and effective approach that provides analgesia, stabilizes vital parameters, and offers comfort to neonates [22-24]. A study that compared the effect of EBM, 25% dextrose, and sterile water on procedural pain in neonates as assessed by PIPP found that EBM significantly reduces procedural pain in neonates, though to a lesser extent as compared to 25% dextrose [23]. In a systematic review, it was concluded that breastfeeding or supplemental breast milk is effective in reducing procedural pain in neonates during minor painful procedures and is preferable to placebo or other non-pharmacological measures. Validated pain scores like Neonatal Infant Pain Scale (NIPS) and Neonatal Facial Coding System (NFCS) were lower in breastfeeding groups compared to controls, although some comparisons with sucrose showed no significant differences. Supplemental breast milk was found to have a lower increase in heart rate when compared to water, and a lower duration of crying when compared to placebo [24].

The clinical relevance of these findings is substantial in proving that both KMC and EBM are cost-effective, accessible interventions suitable for implementation in resource-constrained settings. Additional benefits of KMC, such as improved maternal-infant bonding and neurodevelopmental outcomes, make it a valuable option for routine neonatal care [16,18].

Therefore, in our study, we planned to compare the effect of KMC vs. EBM in reducing procedural pain during adhesive tape removal in low-birth-weight neonates. The study revealed that pain relief efficacy, as assessed by PIPP scores, was comparable between the KMC and EBM groups during adhesive tape removal (p = 0.704). HR and SpO₂, key physiological stress indicators, showed no significant differences between the KMC and EBM groups during both pre- and post-procedure assessments. Similarly, behavioral parameters, such as facial expressions, revealed comparable outcomes between the two groups, corroborating findings from previous literature indicating that both interventions effectively alleviate procedural stress in neonates [14,25].

The only other study that compared these two interventions was by Nanavati et al., which also found that the post-intervention mean PIPP pain score was not significantly different between the KMC and EBM groups (p = 0.62) [14]. Additionally, a key strength of our study lies in the comparison of the change in PIPP scores before and after the intervention, rather than relying solely on post-procedure scores. In our study, we compared the pre-post difference in PIPP scores (mean ± SD) between the KMC group (4.41 ± 1.72) and the EBM group (4.04 ± 1.50), and found that the difference was statistically nonsignificant (p = 0.275). This approach was distinct from previous studies, which primarily compared only the post-procedural PIPP scores. Analyzing the change from baseline helped control for potential confounders, such as pre-existing agitation or discomfort in the neonate, which could otherwise result in falsely elevated baseline pain scores. This strengthened the internal validity of our findings by ensuring that the observed differences in pain responses were attributable to the interventions rather than baseline variability and added a unique dimension to the existing literature on neonatal pain management strategies.

Despite their efficacy, implementing KMC and EBM in NICUs can be challenging. KMC requires parental participation and sufficient space in NICUs, which may not always be feasible in overcrowded settings. EBM, while straightforward to administer, demands strict hygiene protocols to prevent contamination. Standardized training for caregivers and healthcare providers, coupled with education about the benefits of these interventions, is critical for successful implementation.

The primary limitations of our study include the absence of blinding in outcome assessments, a single-center study, reliance on a single assessor without inter-rater reliability checks, and a focus on short-term outcomes. Addressing these limitations in future research could provide a more comprehensive understanding of the relative efficacy and broader implications of KMC and EBM in neonatal care. Future research should focus on multicentric trials with larger, diverse populations to enhance generalizability. Studies are needed to assess the long-term neurodevelopmental and behavioral impacts of KMC and EBM. Comparing these methods with other non-pharmacological interventions such as oral sucrose, non-nutritive suckling, or swaddling, exploring their combined effects, and evaluating cost-effectiveness and scalability in low-resource settings will further guide clinical practice.

## Conclusions

KMC and EBM have both demonstrated proven efficacy in reducing procedural pain during adhesive tape removal in low-birth-weight neonates. Our study found no significant difference between the two methods, reinforcing that both are equally effective non-pharmacological interventions for neonatal pain management. These strategies are simple, safe, cost-effective, and feasible even in resource-limited settings. Their integration into routine neonatal care can significantly improve the quality of care and comfort for neonates.
